# Study of the Optimal Composition and Storage Conditions of the Fricke–XO–Pluronic F–127 Radiochromic Dosimeter

**DOI:** 10.3390/ma15030984

**Published:** 2022-01-27

**Authors:** Michał Piotrowski, Piotr Maras, Sławomir Kadłubowski, Marek Kozicki

**Affiliations:** 1 Department of Mechanical Engineering, Informatics and Chemistry of Polymer Materials, Faculty of Materials Technologies and Textile Design, Lodz University of Technology, 90-543 Lodz, Poland; michal.piotrowski@dokt.p.lodz.pl; 2 Department of Radiotherapy Planning, Copernicus Hospital, 93-513 Lodz, Poland; p.maras@kopernik.lodz.pl; 3 Institute of Applied Radiation Chemistry, Faculty of Chemistry, Lodz University of Technology, 93-590 Lodz, Poland; slawomir.kadlubowski@p.lodz.pl

**Keywords:** Fricke–XO–Pluronic F–127, 3D radiochromic dosimeter, radiotherapy dosimeter, ionizing radiation

## Abstract

This paper presents the results of research on the Fricke–XO–Pluronic F–127 dosimeter. It consists of a Fricke dosimetric solution and xylenol orange (XO), which are embedded in a matrix of poly(ethylene oxide)-*block*-poly(propylene oxide)-*block*-poly(ethylene oxide) (Pluronic F–127). Upon irradiation, Fe^+2^ ions transform into Fe^+3^, forming a colored complex with XO ([XO-Fe]^+3^). The color intensity is related to the dose absorbed. The optimal composition, storage conditions, and radiation-induced performance of the Fricke–XO–Pluronic F–127 dosimeter were investigated. The optimal composition was found to be 1 mM FAS, 50 mM sulfuric acid (H_2_SO_4_), 0.165 mM XO in 25% Pluronic F–127. The basic features of this dosimeter are discussed, such as dose sensitivity, linear and dynamic dose range, stability before and after irradiation, storage conditions, dose response for irradiation with 6 and 15 MV photons, and batch-to-batch reproducibility. The obtained results showed a certain potential of the Fricke–XO–Pluronic F–127 for radiotherapy dosimetry.

## 1. Introduction

The first mentions of 3D radiotherapy based on a standard Fricke dosimetric solution embedded in a polymer matrix come from the 1980s [1,2]. This dosimeter was measured with magnetic resonance imaging. The present focus on the Fricke 3D dosimeter is related to optical computed tomography reading [3]; however, the dosimeter is additionally infused with color forming substances to facilitate optical measurements [4,5,6,7,8].

Numerous studies focused on different aspects regarding Fricke 3D dosimetry, including optimization of gel dosimeter’s formulae, stability, reproducibility, and dose response characterizations by using different radiation sources and reducing the diffusion of Fe^+3^ ions after irradiation [9,10,11,12].

In order for the dosimeter to be used for three-dimensional measurements of the dose distribution and thus maintain the changes in 3D due to absorbed dose after non-uniform irradiation, the radiation-sensitive reagents must be embedded in a 3D matrix. Various matrices were tested, such as agarose [13,14,15,16,17,18], gelatin [19,20,21,22], and poly(vinyl alcohol) (PVA) [23,24].

In addition to the above-mentioned matrices for the preparation of Fricke 3D gel dosimeters, an alternative matrix has been proposed. It is based on poly(ethylene oxide)-*block*-poly(propylene oxide)-*block*-poly(ethylene oxide) (Pluronic F–127) [25], which creates physical gels with a high degree of transparency and colorlessness, allows to prepare a Fricke gel dosimeter at low temperatures due to the phase behavior of the Pluronic F–127; it is stable over a wide temperature range and has been proven to be non-toxic (approved by the Food and Drug Administration, FDA, Silver Spring, MD, USA) [26,27]. Also, Pluronic F–127 has been used for numerous applications such as for the preparation of micelles as a drug delivery system for photodynamic therapy [28] or nanoparticles as antioxidant carriers to stabilize polymers [29]. Besides, as a non-toxic co-polymer, Pluronic F–127 was widely used for the encapsulation of living cells and in other biomaterials research [30]. So far, there have been several gel dosimeters for 3D radiotherapy developed on the basis of Pluronic F–127 [25,30,31,32,33,34,35,36,37,38].

To this point, one attempt has been made to prepare and test a Fricke dosimeter with a Pluronic F–127 matrix [39] in accordance with the literature suggestions for composition. In this work, however, only the issue related to the possible anisotropic diffusion of [XO-Fe]^+3^ complexes formed after irradiation in Fricke–XO–Pluronic F–127 relative to the Fricke−XO−gelatine dosimeter was examined. In turn, in the present work, the optimal composition was searched for by the Fricke–XO–Pluronic F–127 dosimeter. These included studies of the dosimeter stability for a selected composition in order to obtain the conditions in which the dosimeter should be kept after preparation and irradiation, the study of the effect of air on dosimeter stability and dose response, and the study of the effect of component concentration on dosimeter dose performance. The optimal composition, storage conditions and its main dose-response features have been found.

## 2. Materials and Methods

### 2.1. Preparation of Dosimeter

Fricke–XO–Pluronic F–127 3D radiotherapy gel dosimeter samples were prepared using 25% *w*/*w* Pluronic F–127 (Pluronic^®^ F–127, Sigma-Aldrich, BioReagent, Saint Louis, MO, USA) as a gel matrix, 50 mM sulfuric acid (H_2_SO_4_, Chempur, Piekary Śląskie, Poland), 0.01, 0.1, 0.5, 1, and 5 mM ammonium iron (II) sulphate hexahydrate ((NH_4_)_2_Fe(SO_4_)_2_·6H_2_O, FAS, Chempur, Piekary Śląskie, Poland) and 0.03, 0.08, 0.165, 0.3, and 0.5 mM xylenol orange (XO, Sigma-Aldrich, Saint Louis, MO, USA). The Pluronic F–127 solution was prepared as described elsewhere [25]. Then, all reagents (except Pluronic F–127) were dissolved in distilled water and added to the Pluronic F–127 solution. The mixing of the final solution was gentle in order not to produce a Pluronic F–127 foam as Pluronic F–127 is a surfactant with excellent foaming properties. After preparation, the solution was transferred to plastic UV-Vis spectrophotometric cuvettes with an optical path of 1 cm.

### 2.2. Storage Conditions of Fricke–XO–Pluronic F–127 

The Fricke–XO–Pluronic F–127 dosimeter was prepared as described in Section 2.1. Dosimetric samples in plastic UV-Vis cuvettes (optical path 1 cm) were stored in a refrigerator (~4 °C), a cabinet without access to daylight (room temperature around 21 °C) and on a window sill, exposed to daylight and temperature fluctuations of 21 ± 5 °C. The samples were periodically measured by using a UV-Vis spectrophotometer (Jasco V-530, Tokyo, Japan) (Section 2.5).

The storage conditions of the dosimeter were also examined in relation to the access of oxygen (air) to the dosimeter. For this purpose, some of the cuvettes were completely filled, covered with adapted plastic caps, which were additionally wrapped in Parafilm^®^. Dosimeters in the cuvettes prepared in this way were treated as with no infusion of free oxygen (the plastic of the cuvettes could be to some extent oxygen permeable, which was not assessed in this study). On the other hand, some cuvettes were incompletely filled (approximately 90% of the cuvette volume was filled) and were covered with the same plastic caps, but were not wrapped in Parafilm^®^. Dosimeters in such cuvettes were considered to have access to oxygen during irradiation and storage (called also as the samples with reduced/hindered oxygen infusion). Finally, some samples were covered with just one layer of Parafilm^®^ to protect them against water evaporation. They were treated as the samples with free oxygen infusion.

### 2.3. Irradiation of Samples

Samples of the Fricke–XO–Pluronic F–127 dosimeter prepared as described in Section 2.1 and Section 2.2 were irradiated with two sources of ionizing radiation: a technical accelerator (ELU 6-E Elektronika, Moscow, USSR; results in Section 3.1, Section 3.2, Section 3.3) and a medical accelerator (Clinac 2300 CDS, Varian, Palo Alto, CA, USA; results in Section 3.4). In the case of the technical accelerator, the samples were irradiated in air and they were positioned perpendicular to the electron beam (6 MeV). The duration of a single pulse was 17 ns, and the frequency was 20 Hz. The dose rate ranged from 0.7 to 1.0 Gy/s (precision for dose rate measurements was ±10%). In the case of a medical accelerator (TrueBeam, Varian, Palo Alto, CA, USA), the samples were placed 5 cm below the water surface in a water phantom (Blue Phantom 2, IBA Dosimetry GmbH, Schwarzenbruck, Germany; temperature ~19–20 °C) and irradiated with a 6 or 15 MV photon beam (dose rate: X6 0.101 Gy/s; X15 0.1094 Gy/s; field size 20 × 20 cm^2^, SSD 95 cm). The samples were irradiated in the accelerator isocenter perpendicularly to the beam axis. Dose values were obtained on the basis of dosimetric measurements using a CC04 ionizing chamber (IBA Dosimetry GmbH, Schwarzenbruck, Germany) and Dose-1 electrometer (IBA Dosimetry GmbH, Schwarzenbruck, Germany); measurements in the Blue Phantom 2.

### 2.4. Stability of Samples after Irradiation

In order to determine the stability of the Fricke–XO–Pluronic F–127 over time after irradiation, a series of oxygen-permeation protected samples were prepared (Section 2.1 and Section 2.2). The prepared samples were stored in a fridge at about 4 °C. They were irradiated 24 h after their preparation with the aid of an electron accelerator as specified in Section 2.3 (ELU 6-E Elektronika, Moscow, USSR). Then, UV-Vis spectrophotometric measurements were made up to 168 h after irradiation. The samples were stored in a cabinet at a temperature of approximately 21 °C between measurements.

### 2.5. UV-Vis Measurements

For absorbance assessment of the Fricke–XO–Pluronic F–127, UV–vis spectrophotometry was applied. For this purpose, Jasco V-530 instrument (Tokyo, Japan) was used, and spectra were recorded over the wavelength of 190–700 nm with 1 nm resolution. Each sample was measured with a reference to air since the plastic of the cuvette contributed marginally to UV-Vis spectrum of the dosimeter (the mean absorbance of the plastic cuvette used is equal to 0.069 ± 0.007 for the wavelength range 320–700 nm, and 0.091 ± 0.005 for the wavelength range 320–350 nm). Thus, whenever absorbance or optical density (Δµ) is discussed, it is understood as absorbance or optical density of the Fricke–XO–Pluronic F–127 with a plastic cuvette. The optical density was calculated as follows: Δµ = (ln(10)/x) × (AI–AU), where x = 1 cm is the length of the light path through the vial, AI is the measured absorbance of the irradiated sample and AU is the absorbance of the non-irradiated sample; in both cases, the absorbance values at 585 nm were taken for calculations.

## 3. Results and Discussion

### 3.1. Stability of Non-Irradiated Fricke–XO–Pluronic F–127 

**Figure 1:**
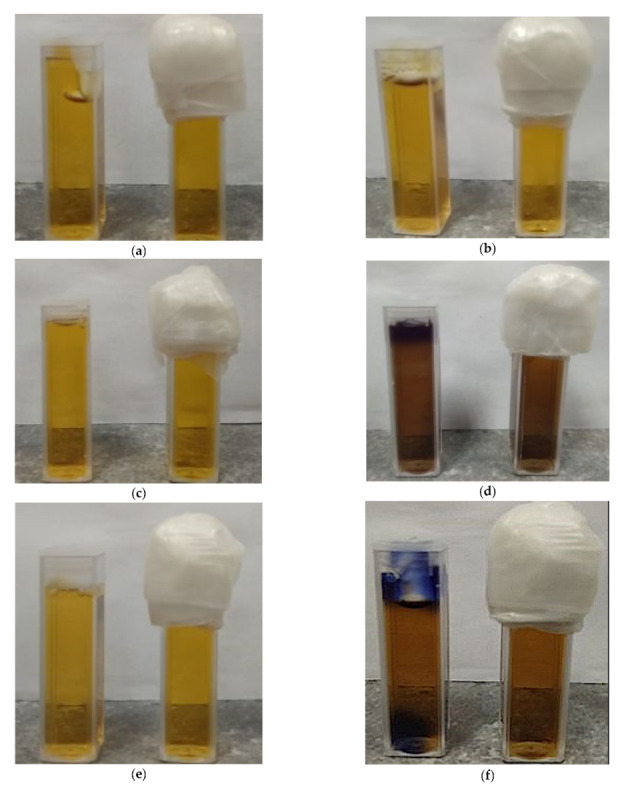
Photographs of the stability of Fricke–XO–Pluronic F–127 gels (FAS: 0.5 mM, H_2_SO_4_: 50 mM, XO: 0.165 mM, Pluronic F–127: 25%) stored: in a refrigerator at around 4 °C without access to daylight (**a**,**b**); on a window sill exposed to daylight and temperature changes (**c**,**d**); in a cabinet protected from daylight and a room temperature of around 21 °C (**e**,**f**). In the photographs, the cuvettes on the left side are those of hindered access to free oxygen from the air, filled to about 90% of the cuvette volume covered with a plastic cap (hindered access of free oxygen from the air but 10% of the volume covered with air), whereas on the right side are the cuvettes filled in completely and tightly closed (assuming no oxygen infusion). The photographs in the left column (**a**,**c**,**e**) were taken immediately after the dosimetric samples were prepared, while the photos in the right column (**b**,**d**,**f**) were taken 24 h after the preparation. The purple color at the top of the cuvette in Figure 1 (**f**) is a typical phenomenon seen in the vicinity of air bubbles during prolonged storage.

**Figure 2:**
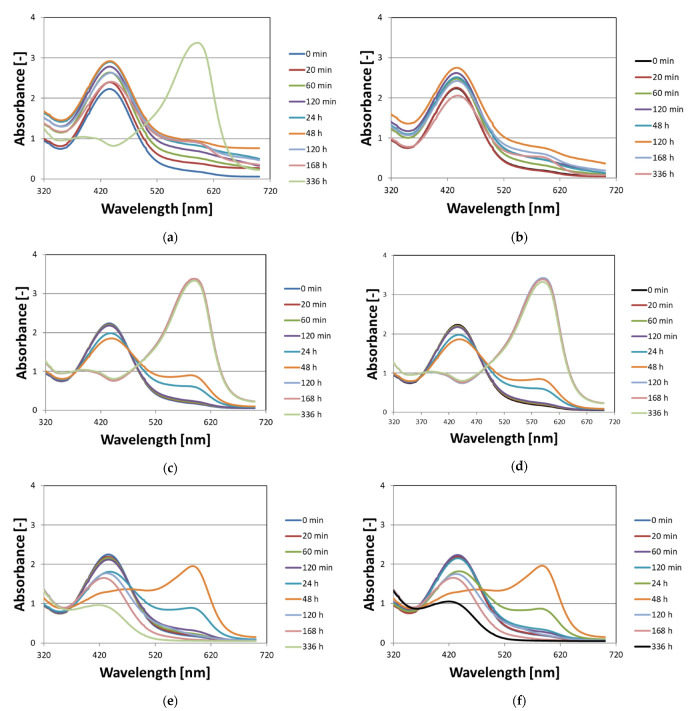
Stability of Fricke–XO–Pluronic F–127 gels (FAS: 0.5 mM, H_2_SO_4_: 50 mM, XO: 0.165 mM, Pluronic F–127: 25%) stored: in a fridge at around 4 °C without access to daylight (**a**,**b**); in a cabinet without access to daylight and room temperature of around 21 °C (**c**,**d**); on a window sill exposed to daylight and temperature variations (**e**,**f**). Graphs (**a**,**c**,**e**) correspond to the Fricke–XO–Pluronic F–127 samples in cuvettes filled to about 90% volume of the cuvette covered with a plastic cap (hindered access of free oxygen from air but with 10% volume covered by air), whereas graphs (**b**,**d**,**f**) correspond to the cuvettes filled in completely and tightly closed (assumed no oxygen infusion).

This part of the work aimed to answer the following questions related to non-irradiated Fricke–XO–Pluronic F–127 dosimeter: (i) what is the influence of the storage method on the stability of the dosimeter and (ii) how the availability of oxygen (air) affects the stability of the dosimeter. To answer the questions, Fricke–XO–Pluronic F–127 samples in cuvettes filled to about 90% volume of the cuvette covered with a plastic cap (hindered access of free oxygen from air but with 10% volume covered by air), and in cuvettes filled in completely and tightly closed (assumed no oxygen infusion) were stored at room temperature with protection from daylight, at room temperature with access to daylight and at low temperature (4 °C) without access to daylight. The samples were analyzed with the aid of UV-Vis spectrophotometry. The obtained results are presented in the form of photographs (Figure 1) and absorbance spectra (Figure 2) of the dosimetric samples over time of storage.

The analysis of the Fricke–XO–Pluronic F–127 photographs (Figure 1) led to the following conclusions: (i) samples of all variants are transparent, uniform in color and yellow just after preparation (Figure 1a,c,e), (ii) all samples change optically over time of storage, regardless of storage conditions, (iii) the highest stability was achieved for the samples stored in a refrigerator at a temperature of about 4 °C in the dark (Figure 1a,b); these samples, irrespective of the method of covering the cuvettes and the access of oxygen, were stable for at least 120 h after preparation. After this time, the cuvettes filled in completely and tightly covered with a cap and Parafilm^®^ turned dark brown, while the dosimeter in the second cuvette turned very dark navy blue, which was observed 168 h after preparation (the photographs for longer storage time are not shown), (iv) the dosimeter samples stored at room temperature (Figure 1c–f) turned dark brown just 24 h after preparation, regardless of the method of coating (oxygen access) and exposure to daylight. However, these samples, when exposed to or protected from daylight, behaved differently over a longer period of time. Samples exposed to daylight began to bleach after reaching a maximum dark brown color, resulting in a pale yellow transparent gel dosimeter. On the other hand, samples that were protected from daylight continued to convert to very dark navy blue, which was observed up to 336 h after preparation (not shown in Figure 1). The observations regarding the optimal storage conditions (low temperature, protection from daylight) for non-irradiated Fricke–XO–Pluronic F–127 are similar to those for Fricke−XO−gelatine published elsewhere [9]. However, storing the Fricke−XO−gelatin for two weeks resulted in the growth of the fungus [9]. This was not observed with Fricke–XO–Pluronic F–127 that had been refrigerated for about 12 months.

**Figure 3:**
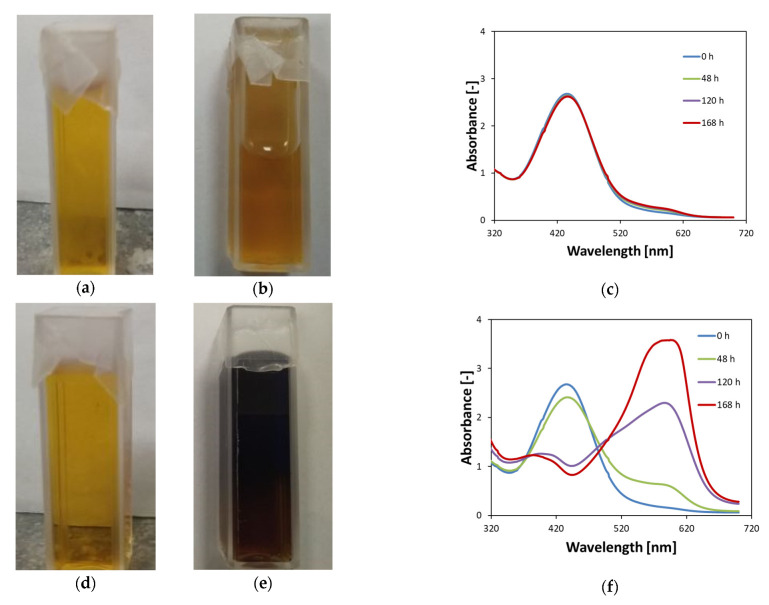
Stability of Fricke–XO–Pluronic F–127 gels in cuvettes with access to oxygen—only slightly closed with Parafilm^®^ (0.5 mM FAS, 50 mM H_2_SO_4_, 0.165 mM XO, 25% Pluronic F–127): (**a**–**c**) are for the dosimeter stored in a refrigerator at around 4 °C without access to daylight, whereas (**d**–**f**) relate to the dosimeter samples stored in a cabinet without access to daylight at a room temperature around 21 °C. Photographs (**a**,**b**) as well as (**d**,**e**) were taken immediately after preparation and after 120 h after preparation, respectively. The samples in (**b**,**e**) are placed horizontally on the table to illustrate the flow of the dosimeter solution for (**b**) immediately after being removed from the refrigerator.

The absorbance spectra of the Fricke–XO–Pluronic F–127 gel dosimeter recorded during storage (Figure 2) correspond to observations with the naked eye in the photographs in Figure 1. Figure 2a,c,e is for the Fricke–XO–Pluronic F–127 with oxygen access, while Figure 2b,d,f is for the dosimeter without oxygen access (as explained in the caption in Figure 2). However, Figure 2a,b is for the dosimeter stored in a refrigerator, Figure 2c,d is for the dosimeter stored in a cabinet at room temperature and Figure 2e,f is for the dosimeter stored at room temperature but exposed to daylight. Generally, for the freshly prepared Fricke–XO–Pluronic F–127 gel, there is a characteristic band at 440 nm. This band evolves during storage, which corresponds to the color changes of the dosimeter. Spectral analysis showed that the Fricke–XO–Pluronic F–127 is stable for the longest period of time when refrigerated and without oxygen access (Figure 2b). In this case, there is a slight increase in the intensity of absorbance at 440 nm up to 120 h after preparation, and then a decrease to 336 h after preparation. The instability of Fricke–XO–Pluronic F–127 can be well observed by the band build-up at 585 nm. This band corresponds to the formation of [XO-Fe]^+3^ [10,40,41]. The intensity of this band is low for a refrigerated dosimeter (Figure 2b), but is significant for all other cases discussed in Figure 2. If the same dosimeter, which is stored in the refrigerator, has some access to oxygen, the intensity of the band at 585 nm is seven times greater than that for the sample without access to oxygen 336 h after preparation (Figure 2a). An interesting evolution of the Fricke–XO–Pluronic F–127 spectra was observed for the samples stored at room temperature, exposed (Figure 2e,f) and unexposed (Figure 2c,d) to daylight. For the samples without access to daylight, the band at 585 nm increases to 336 h after preparation during storage. In contrast, for samples exposed to daylight, this band at 585 nm increases to 48 h after preparation, and then drops down to almost 0 [−] absorbance; at the same time, there is a hypsochromic shift for the band at 440 nm (Figure 2e,f)—the dosimeter pales. Overall, this part of the study showed that Fricke–XO–Pluronic F–127 should be refrigerated at a low temperature. Here, the variants were considered where the dosimeter was tightly capped to eliminate oxygen infusion, and the variant where the dosimeter had some access to oxygen from the air; the samples were closed with the cap and some air gap was left under the cap were considered. The obtained results suggest that the dosimeter should be well protected against air when stored in a refrigerator. However, the option with free access of the dosimeter to oxygen from the air was not considered. The relevant observations are presented below (Figure 3).

Figure 3 shows the stability of Fricke–XO–Pluronic F–127 gels for a dosimeter stored in a refrigerator at approximately 4 °C and in a cabinet at room temperature (both protected from daylight); the samples were gently covered with one layer of Parafilm^®^, which was considered to allow the free infusion of oxygen (air) into the substances of the dosimeter. Cooling the dosimeter with oxygen has been found to aid in its storage for approximately 168 h after preparation. It seems that up to 120 h, there are no visible optical changes in the dosimeter; between 120 and 168 h after preparation, a slight increase in the intensity of the 585 nm band indicates the formation of [XO-Fe]^+3^ at a low concentration. Nevertheless, such a dosimeter can be considered suitable for measuring the radiation dose. In contrast, keeping the dosimeter open to oxygen infusion at room temperature (Figure 3d–f) causes rapid optical changes and a very high intensity 585 nm band to appear 48 h after preparation. Summarizing the observations related to Figure 2 and Figure 3, Fricke–XO–Pluronic F–127 after preparation should be kept in a refrigerator at a low temperature and either gently covered with, e.g., Parafilm^®^ preventing water evaporation but allowing oxygen infusion or filled in completely and tightly covered to prevent oxygen infusion.

### 3.2. Stability of Irradiated Fricke–XO–Pluronic F–127 

**Figure 4:**
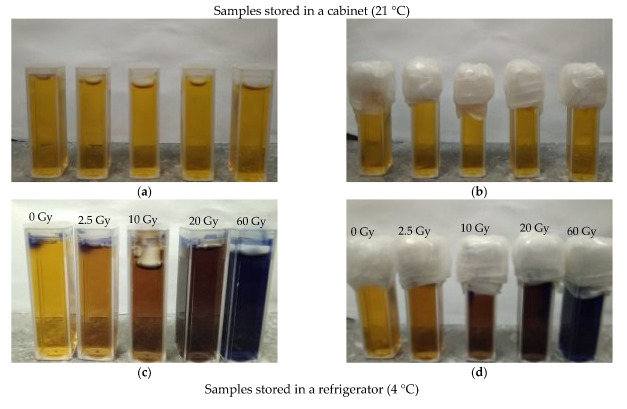

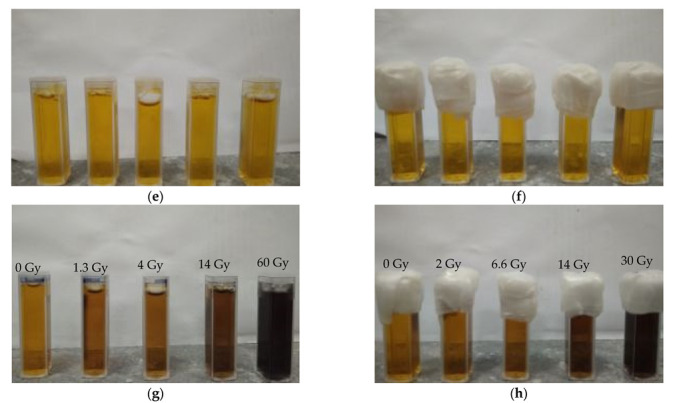
Photographs of Fricke–XO–Pluronic F–127 samples before (**a**,**b**,**e**,**f**) and after (**c**,**d**,**g**,**h**) irradiation for the samples stored in a refrigerator (4 °C) and in a cabinet (21 °C) in the dark: (**a**,**c**,**e**,**g**) correspond to the samples with hindered oxygen access, whereas (**b**,**d**,**f**,**h**) correspond to the samples with no oxygen access.

When Fricke–XO–Pluronic F–127 is exposed to ionizing radiation, it changes from transparent light yellow-brown to dark brown, and at a higher dose to purple. The color intensity increases as the radiation dose increases. Examples of dosimeter samples exposed to radiation are shown in Figure 4. The presented samples were stored after irradiation in a cabinet (21 °C) or in a refrigerator (4 °C) in the dark. The method of covering the samples and the access of oxygen to the substance of the dosimeter were also taken into account in the tests of the samples (Figure 4). In general, samples tightly covered and protected against oxygen infusion appear to be more stable than those with only hindered access to oxygen. This is in line with previous observations. Also, the samples that were refrigerated after irradiation showed higher storage stability than the samples stored in the cabinet (Figure 4a,b compared to Figure 4e,f). The observations regarding the optimal storage conditions (low temperature, protection from daylight) for irradiated Fricke–XO–Pluronic F–127 are similar to those for Fricke−XO−gelatine published elsewhere [9].

If 1D dosimetry of ionizing radiation is being considered, the Fricke–XO–Pluronic F–127 dosimeter may be stored in a refrigerator after irradiation if the reading of this dosimeter is planned for a longer period of time after irradiation. It should be emphasized, however, that lowering the temperature of this dosimeter below room temperature causes the physical hydrogel of the Pluronic F–127 copolymer to melt. On the other hand, low temperature preserves the optical changes of the dosimeter after irradiation for a longer time compared to the dosimeter stored at room temperature. However, if one plans to use the dosimeter as a 3D dosimeter in radiotherapy, keeping it in the refrigerator after inhomogeneous irradiation is pointless. The advantage and purpose of 3D dosimetry is to preserve information about the 3D dose distribution. Therefore, lowering the temperature would melt the dosimeter and, consequently, lose information on the three-dimensional dose distribution. Therefore, the Fricke–XO–Pluronic F–127, intended to serve as a 3D dosimeter, should be stored after irradiation at room temperature in the dark and should be read immediately after irradiation.

**Figure 5:**
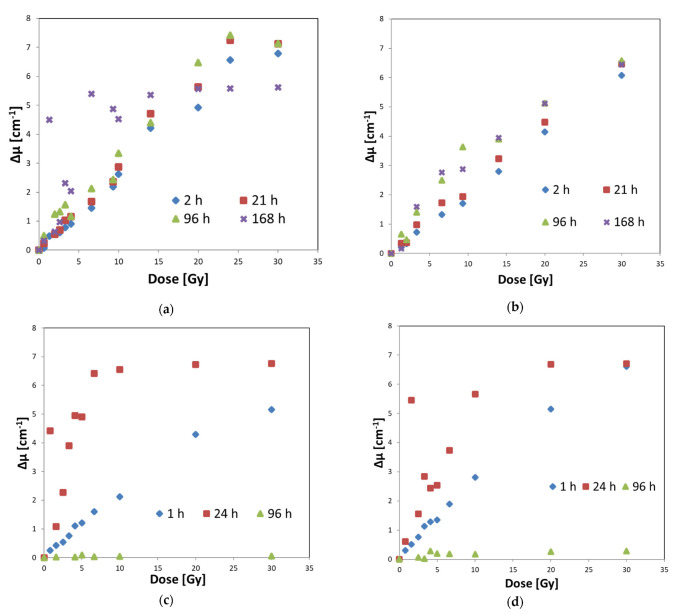
Optical density (Δμ) of Fricke–XO–Pluronic F–127 dosimeter after irradiation for the dosimeter stored in (**a**) a refrigerator for the samples with reduced oxygen access (4 °C), (**b**) in a refrigerator for the samples tightly covered and protected against oxygen infusion (4 °C), (**c**) in a cabinet for the samples with reduced oxygen access (21 °C), and (**d**) in a cabinet for the samples tightly covered and protected against oxygen infusion (21 °C).

The above is confirmed by the results of measurements of the optical density of the irradiated Fricke–XO–Pluronic F–127 shown in Figure 5. The dosimeter reacts to doses by increasing its optical density. The most stable samples were stored in a refrigerator for about 21 h after irradiation (Figure 5a,b). The same samples, but kept in a cabinet at room temperature for about 24 h, changed their optical density much more (Figure 5c,d) than those in the refrigerator. This means that such samples, stored in a cabinet, should be read immediately after irradiation.

Based on the calibration graphs obtained for the relationship between optical density and radiation dose, the basic calibration characteristics were extracted, and the results are presented in Table 1. It can be concluded that: (i) the quasi-linear dose response is up to a dose of about 20 Gy, (ii) dynamic dose response is over 35 Gy (the dose causing saturation of the dosimeter; maximal color change), (iii) the dose sensitivity appears to be comparable regardless of the covering and storage conditions of the dosimeter, and ranges from about 0.22 to 0.29 [Gy^−1^ cm^−1^].

### 3.3. Optimal Composition of Fricke–XO–Pluronic F–127 

**Figure 6:**
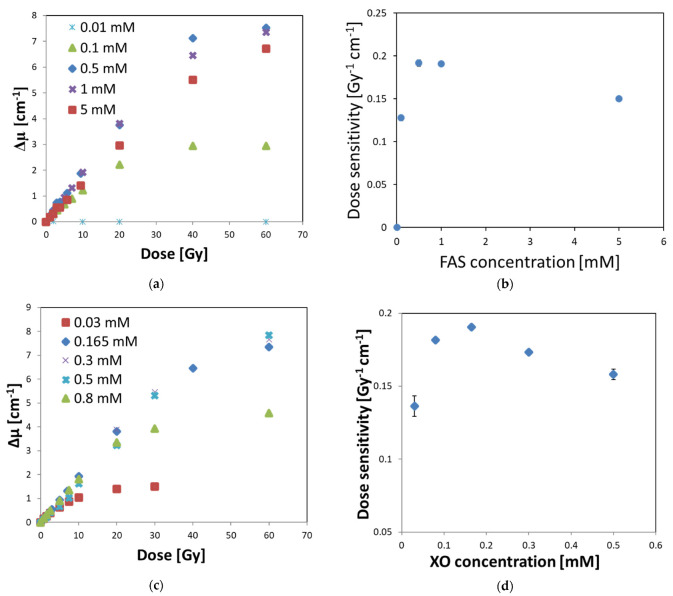
Optical density versus radiation dose and corresponding dose sensitivity versus concentration of FAS (**a**,**b**) or XO (**c**,**d**) for Fricke–XO–Pluronic F–127 gel dosimeter of the composition, as follows: (**a**,**b**) 25% Pluronic F–127, 0.165 mM XO, 50 mM H_2_SO_4_, and x mM FAS (concentration of FAS is given in figure (**a**–**d**) 25% Pluronic F–127, x mM XO, 50 mM H_2_SO_4_, and 1 mM FAS (concentration of XO is given in figure (**c**,**d**). Samples were covered with one layer of Parafilm^®^ (which was considered as free oxygen access), protected from daylight and stored at room temperature. Optical measurements were taken up to 2 h after irradiation. Standard deviation bars for the dose sensitivity values are covered by points in (**b**).

The Fricke–XO–Pluronic F–127 dosimeter consists of a matrix of Pluronic F–127 co-polymer, sulfuric acid, FAS, and XO forming a complex with Fe^+3^ generated from FAS upon irradiation of the dosimeter. While the concentration of Pluronic F–127 was set at 25% after former studies [25] and the concentration of sulfuric acid seems to play a minor role, the concentrations of XO and FAS used by other authors to produce Fricke-based dosimeters [42,43,44] were varied and had an effect on the dose response performance of the dosimeter. It was also interesting to investigate what the optimal concentration of XO and FAS is in the Fricke–XO–Pluronic F–127, which gives the highest dose sensitivity of the dosimeter. Therefore, one concentration of FAS or XO was kept constant and the concentration of the second component in the dosimetric composition was changed, followed by irradiation and UV-Vis measurements to obtain the dependencies of the optical density on the radiation dose. The obtained results are shown in Figure 6.

In Figure 6a, the optical density as a function of radiation dose is presented for FAS of the concentration range of 0.01–5 mM (other components: 25% Pluronic F–127, 0.165 mM XO, 50 mM H_2_SO_4_). On the basis of linear regressions of Δμ = f(dose) for the quasi-linear ranges, the dose sensitivities were identified, corresponding to the slopes of the regressions. The results in Figure 6b indicate that the maximum dose sensitivity is around 0.5–1 mM FAS. Therefore, in the subsequent study, the chosen concentration of FAS was 1 mM and the XO concentration was changed to investigate the optimal one (Figure 6c,d). It occurred that the maximum dose sensitivity was obtained for an XO concentration of 0.165 mM.

The optimal composition of the dosimeter was found to be: 25% Pluronic F–127, 0.165 mM XO, 1 mM FAS, and 50 mM H_2_SO_4_. The dose response is linear up to about 20 Gy (definitely less than 40 Gy), the dynamic dose response is slightly above 60 Gy (maximum dose applied); the system begins to saturate at this dose. The dose sensitivity is 0.191 Gy^−1^ cm^−1^. However, it should be noted that the dose-response characteristics, and thus the dose-sensitivity value, in this part of the study were obtained for a dosimeter irradiated with a technical accelerator, for which the dose rate can be measured with a precision of ±10%; the precision of delivering low doses (in radiotherapy dose range) is definitely lower than that for a medical accelerator. Thus, although the obtained results are reliable and indicate the optimal composition of Fricke–XO–Pluronic F–127, it was necessary to calibrate the dosimeter with a medical accelerator to obtain more accurate data for the dosimeter to be used in radiotherapy.

### 3.4. Calibration of Fricke–XO–Pluronic F–127 with a Medical Accelerator

**Figure 7:**
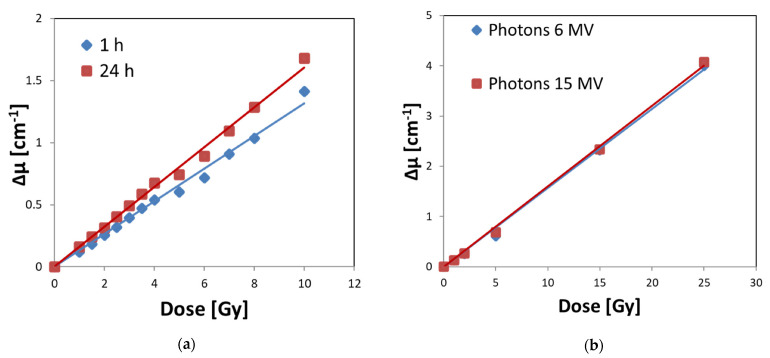
Calibration of Fricke–XO–Pluronic F–127 (25% Pluronic F–127, 0.165 mM XO, 1 mM FAS, 50 mM H_2_SO_4_) expressed as optical density versus radiation dose relations for the dosimeter measured 1 and 24 h after irradiation with 6 MV photons (**a**). In (**b**), the same calibration is presented for the dosimeter irradiated with 6 and 15 MV photons and measured 24 h after irradiation. The samples were irradiated with a medical accelerator (TrueBeam, Varian). Samples in cuvettes were covered with one layer of Parafilm^®^; they were treated with free oxygen infusion.

The optimal Fricke–XO–Pluronic F–127 composition obtained in this work was irradiated with a medical accelerator to test: (i) its dose response and dose sensitivity, (ii) batch reproducibility, (iii) the effect of 6 and 15 MV photon irradiation on dose response, and (iv) temporary stability when stored at room temperature in the dark. The results are presented in Figure 7. After analyzing the figure, the following conclusions were drawn. The dosimeter is unstable and will change during storage. Therefore, it should be measured shortly after irradiation (Figure 7a). This is in line with previous observations. The dose sensitivity derived from the regression of the linear optical density (Δμ) as a function of the radiation dose is 0.1316 ± 0.0022 [Gy^−1^ cm^−1^] (R^2^ = 0.996) and 0.1607 ± 0.0021 [Gy^−1^ cm^−1^] (R^2^ = 0.998), for calibrations obtained 1 and 24 h, respectively, after irradiation (Figure 7a). The dose response is linear up to 25 Gy (the maximum dose used in this study) and the dosimeter responds to irradiation in the same way over the range of 6–15 MV photons (Figure 7b). The dose sensitivity obtained for 6 and 15 MV photons (24 h after irradiation) is 0.1572 ± 0.0031 [Gy^−1^ cm^−1^] (R^2^ = 0.998) and 0.1601 ± 0.0024 [Gy^−1^ cm^−1^] (R^2^ = 0.999), respectively. Thus, if the dose sensitivities of the samples measured 24 h after irradiation (Figure 7a,b) are compared, it can be concluded that they are the same within the standard deviation and oscillate around 0.16 [Gy^−1^ cm^−1^]. It also means that the used method of preparation and storage of Fricke–XO–Pluronic F–127 results in reproducible batches of dosimeter in terms of their dose response.

## 4. Conclusions

In this work, Fricke–XO–Pluronic F–127 was examined in terms of optimal composition, stability before and after irradiation, dose response features, and batch-to-batch repeatability of manufacturing. The optimal composition was found to be 1 mM ammonium iron (II) sulphate hexahydrate ((NH_4_)_2_Fe(SO_4_)_2_·6H_2_O, FAS), 50 mM sulfuric acid (H_2_SO_4_), and 0.165 mM xylenol orange (XO) in 25% Pluronic F–127. The dosimeter responded to irradiation with a medical accelerator with the following features: (i) linearity of up to 25 Gy (maximum dose applied), (ii) dose sensitivity of about 0.1316 ± 0.0022 Gy^−1^ cm^−1^, (iii) the same dose response to irradiation with 6 and 15 MV photons, (iv) manufacturing process led to repeatable dosimeters as occurred after the same dose response by three batches. Moreover, the storage stability of the dosimeter is improved if before or after irradiation is kept in a refrigerator, either in containers filled in completely and tightly closed to prevent from oxygen infusion or with free oxygen infusion, in the dark. Because of this, the dosimeter in a refrigerator was stable before irradiation for about 120 h and after irradiation for about 24 h. If, however, the storage in a refrigerator after irradiation is not possible, it should be kept in the dark at room temperature. In such a case, the dosimeter should be read after irradiation preferably within the first 2 h. Judging by the results for irradiation with a technical accelerator, the dynamic dose response of the dosimeter can be slightly over 60 Gy. In conclusion, the Fricke–XO–Pluronic F–127 have the features that make it feasible for application in radiotherapy. However, it should be emphasized that the reading of the dosimeter is done shortly after irradiation (when used as a 3D dosimeter) not only due to its poor storage stability, but also due to likely anisotropic diffusion of the irradiated part over time of storage under gravitational force. The anisotropic diffusion was shown recently in another work [39] not only for Fricke–XO–Pluronic F–127 but also for Fricke−XO−gelatine dosimeter. Fast scanning of the Fricke-based 3D dosimeters with nonzero [XO-Fe]^+3^ diffusion coefficients after irradiation should eliminate problems related to storage stability and diffusion anisotropicity of irradiated regions.

## Figures and Tables

**Table 1:** Basic calibration features of the Fricke–XO–Pluronic F–127 for the samples measured with UV-Vis spectrophotometry, up to 2 h after irradiation. The values stem from the calibration graphs of optical density (Δμ) vs. radiation dose (Figure 5).

Oxygen (Air) Access	Dose Sensitivity [Gy^−1^ cm^−1^]	R^2^ [-]	Quasi-Linear Dose Range [Gy]	Dynamic Dose Range [Gy]
Samples stored in the dark in a refrigerator (4 °C)				
No oxygen access	0.221 ± 0.004	0.997	20	~35 Gy or higher
Hindered oxygen access	0.291 ± 0.009	0.990	20	
Samples stored in the dark in a cabinet (21 °C)				
No oxygen access	0.268 ± 0.006	0.995	20	~35 Gy or higher
Hindered oxygen access	0.219 ± 0.004	0.996	20	

## Data Availability

The data supporting the reported results are not stored in any publicly archived datasets. The readers can contact the corresponding author for any further clarification of the results obtained.
